# Temporal turnover and fire severity shape postfire beetle (Coleoptera) diversity at a forest range edge

**DOI:** 10.1093/jisesa/ieag101

**Published:** 2026-10-20

**Authors:** Briseida E Yáñez, Jane E Strobel, Valarie L Gabbard, Karalee A Corbeil, Robert M Plowes, Lawrence E Gilbert, Kenneth P Wray, Steven Gibson, Kelsey Meisenhelder, Alex Wild, Rossana Maguiña-Conde

**Affiliations:** Stengl Lost Pines Biological Station, Biodiversity Center, Department of Integrative Biology, The University of Texas at Austin, Smithville, TX, USA; Stengl Lost Pines Biological Station, Biodiversity Center, Department of Integrative Biology, The University of Texas at Austin, Smithville, TX, USA; Stengl Lost Pines Biological Station, Biodiversity Center, Department of Integrative Biology, The University of Texas at Austin, Smithville, TX, USA; Stengl Lost Pines Biological Station, Biodiversity Center, Department of Integrative Biology, The University of Texas at Austin, Smithville, TX, USA; Brackenridge Field Laboratory, Biodiversity Center, Department of Integrative Biology, The University of Texas at Austin, Austin, TX, USA; Brackenridge Field Laboratory, Biodiversity Center, Department of Integrative Biology, The University of Texas at Austin, Austin, TX, USA; Stengl Lost Pines Biological Station, Biodiversity Center, Department of Integrative Biology, The University of Texas at Austin, Smithville, TX, USA; Brackenridge Field Laboratory, Biodiversity Center, Department of Integrative Biology, The University of Texas at Austin, Austin, TX, USA; Stengl Lost Pines Biological Station, Biodiversity Center, Department of Integrative Biology, The University of Texas at Austin, Smithville, TX, USA; Stengl Lost Pines Biological Station, Biodiversity Center, Department of Integrative Biology, The University of Texas at Austin, Smithville, TX, USA; University of Texas Insect Collection, Biodiversity Center, Department of Integrative Biology, The University of Texas at Austin, Austin, TX, USA; Stengl Lost Pines Biological Station, Biodiversity Center, Department of Integrative Biology, The University of Texas at Austin, Smithville, TX, USA

**Keywords:** habitat heterogeneity, pyrophilous, saproxylic, succession

## Abstract

In recent decades, wildfires have increased in frequency and severity, reshaping forest structure and biodiversity. Yet it remains unclear whether postfire insect assemblages are driven primarily by fire severity, vegetation structure, or broader temporal community turnover, particularly in fragmented range-edge forests. We investigated flight-active beetle (Coleoptera) assemblages 2 years after a 2022 wildfire in the Lost Pines region of Texas, an isolated loblolly pine (*Pinus taeda* L.) forest at the species’ western range margin. Using historical data from 2011, we evaluated temporal turnover and compared diversity and assemblage composition between contemporary burned and unburned sites. We also quantified fire severity and vegetation structure to analyze their effects on richness and abundance. Contemporary and burned sites supported higher richness and abundance than historical and unburned sites, although evenness declined as opportunistic species became dominant. Beetle assemblage differed significantly between sampling periods and burn status, indicating substantial temporal turnover amplified by wildfire. Fire severity showed strong positive associations with richness and abundance, whereas vegetation structure played a secondary but important role. Richness and abundance increased with woody plant richness, tree diversity, tree size, live tree density, and shrub richness. In contrast, dead tree variables were generally weak or negative predictors, suggesting that the influence of fire-generated deadwood may be transient and strongest shortly after disturbance. Together, these findings indicate that postfire flight-active beetle assemblages in the Lost Pines are shaped by the interaction of long-term temporal turnover and fire-driven successional dynamics, with fire severity acting as the dominant driver of community change.

## Abstract (es)

Los incendios forestales aumentan en frecuencia y severidad, modificando la estructura y biodiversidad de los bosques. Sin embargo, desconocemos si los ensamblajes de insectos post-incendio están determinados por la severidad del fuego, la estructura de la vegetación, o el recambio temporal de las comunidades, especialmente en bosques fragmentadosy aislados. Investigamos las comunidades de escarabajos activos en vuelo (Coleoptera) 2 años después de un incendio ocurrido en 2022 en la región de Lost Pines, Texas, en un bosque aislado de pino (*Pinus taeda* L.). Utilizando datos históricos de 2011, evaluamos el recambio temporal y comparamos la diversidad y composición de los ensamblajes entre sitios contemporáneos quemados y no quemados. Además, cuantificamos la severidad del incendio y la estructura vegetal para analizar sus efectos sobre la riqueza y abundancia de escarabajos. Los sitios contemporáneos y quemados presentaron mayor riqueza y abundancia que los sitios históricos y no quemados, aunque la equitatividad disminuyó debido a la dominancia de especies oportunistas. La composición de los ensamblajes difirió significativamente entre periodos de muestreo y condiciones de quema, indicando un importante recambio temporal amplificado por el incendio. La severidad del fuego mostró asociaciones positivas con la riqueza y abundancia, mientras que la estructura vegetal desempeñó un papel secundario, aunque importante, sobre todo la riqueza de plantas leñosa, la diversidad de árboles, la densidad de árboles vivos y la riqueza de arbustos. En conjunto, estos resultados indican que los ensamblajes post-incendio están determinados por la interacción entre el recambio temporal y las dinámicas sucesionales impulsadas por el fuego.

## Introduction

Severe drought and anthropogenic land-use changes are intensifying the frequency, extent, and severity of wildfires across forested ecosystems worldwide (Wasserman and Mueller 2023). These fires can fundamentally reshape forest structure, nutrient availability, and habitat heterogeneity—key factors that directly influence the diversity, composition, and resilience of forest-dwelling communities (Turner et al. 1997, Johnstone et al. 2016). At the same time, forest communities undergo continuous temporal turnover in species composition independent of disturbance (Salomão et al. 2020), complicating attribution of observed change to fire alone. Yet few studies have jointly examined how interacting environmental gradients (fire severity and forest structure) shape postfire community responses through both disturbance effects and background temporal turnover, particularly in fragmented forest landscapes.

Beetles (Coleoptera) provide an effective lens for examining these dynamics because of their immense taxonomic and functional diversity and their sensitivity to postfire habitat conditions (McGeoch 1998, Bouchard et al. 2011, Ulyshen 2011). With over 400,000 described species globally, beetles occupy nearly every terrestrial and freshwater habitat, fulfilling crucial roles in decomposition, herbivory, predation, and pollination (Bouchard et al. 2011, Ulyshen 2011). Their ecological breadth and frequent associations with specific microhabitats make them reliable indicators of ecosystem health, particularly in fire-affected landscapes (McGeoch 1998). Fire alters beetle habitat availability through changes in deadwood, vegetation regrowth, and structural heterogeneity, which can benefit saproxylic and phytophagous species (Moretti et al. 2004, Saint-Germain et al. 2004), or, under severe conditions, reduce habitat complexity and host plant availability (Paquin and Coderre 1997, Swengel 2001). These contrasting outcomes highlight fire severity as a key driver of beetle community dynamics and a critical factor in addressing knowledge gaps in insect responses to fire (Mason et al. 2021, 2023).

Studies have documented a range of postfire responses in beetle diversity and assemblage composition. Some report short-term increases in both species richness and abundance (Hyvärinen et al. 2006, Fredriksson et al. 2020, Gutowski et al. 2020, Lawhorn et al. 2025), while others find higher richness without corresponding changes in abundance (Mason et al. 2021). These increases are most commonly observed among pyrophilous (Bentz et al. 1991, Wikars 1997, Kouki et al. 2012, Gutowski et al. 2020) and saproxylic taxa (Hyvärinen et al. 2009, Lawhorn et al. 2025). Despite these general patterns, responses remain variable across systems, highlighting the importance of study design in understanding fire effects on beetle communities.

Most studies have assessed fire effects by comparing burned and unburned areas or by tracking postfire succession over time (Wikars 1997, Hyvärinen et al. 2009, Kouki et al. 2012, Fredriksson et al. 2020, Gutowski et al. 2020, Lawhorn et al. 2025). Burned–unburned comparisons allow contemporaneous sampling that minimizes temporal variation, but they may be confounded by spatial heterogeneity among sites. In contrast, studies of postfire succession provide insight into recovery trajectories but often lack prefire baselines for evaluating ecological change. Less commonly, historical (prefire) datasets are combined with contemporary postfire sampling to assess long-term community change, although this approach may still confound fire-driven effects with background temporal turnover. Distinguishing these processes remains challenging because insect communities can reorganize substantially over time independent of disturbance (Salomão et al. 2020), and historical datasets suitable for such analyses are rare due to the unpredictability of wildfire. Integrating historical baselines with contemporary postfire sampling nevertheless provides a valuable framework for evaluating how disturbance-driven succession differs from broader temporal change in beetle communities.

Beyond these methodological limitations, the majority of beetle-fire research has taken place within the core of species’ geographic distributions, leaving a significant gap in understanding how beetle communities respond to fire at the edges of their ranges. Peripheral populations often experience heightened climatic and ecological stress, which may amplify their vulnerability to disturbance or result in divergent ecological responses (Hampe and Petit 2005). Therefore, studying insect communities in edge-of-range environments is critical for anticipating how they will respond to ongoing environmental change.

In addition, few studies have explicitly considered how postfire vegetation structure mediates fire effects on beetle assemblages (such as Boulanger et al. 2010, 2013, Hjältén et al. 2017), likely because most research has focused on prescribed fires (Niwa and Peck 2002, Nunes et al. 2006, Hyvärinen et al. 2009, Kouki et al. 2012 except for Fredriksson et al. 2020, Gutowski et al. 2020, Lawhorn et al. 2025), which generally produce less structural change than wildfires. The relationship between beetle assemblages and postfire vegetation legacies remains largely speculative, despite growing evidence that forest age, size, canopy cover, and habitat diversity strongly influence beetle diversity and assemblage composition in non-fire contexts (Wende et al. 2017, Traylor et al. 2022). Structural characteristics—like woody plant density—influence understory microclimates, resource availability, and habitat complexity, while woody plant richness affects the diversity of host plants and substrate types available to beetles (Boulanger et al. 2010, Ulyshen 2011, Seibold et al. 2016, Lettenmaier et al. 2022, Sbaraglia et al. 2025). These vegetation attributes may act independently or interact with fire severity to shape beetle assemblages, yet their roles remain underexplored (Perlík et al. 2023).

The Lost Pines region of central Texas provides an ideal system for examining these dynamics. This relict population of loblolly pine (*Pinus taeda* L., Pinales: Pinaceae), geographically isolated from the species’ main southeastern US distribution, occupies a transition zone where pine forest intergrades with post oak (*Quercus stellata* Wangenh., Fagales: Fagaceae) woodlands. The region’s drier climate, sandy soils, and fragmented landscape create a unique ecological context at the western edge of *P. taeda*’s range. Decades of fire suppression and historical logging in the Lost Pines region have altered forest structure and increased fuel loads, influencing the intensity and spatial patterns of recent wildfires (Brown et al. 2014, Stambaugh et al. 2017) and potentially modifying beetle community resilience. Furthermore, the region isolation and fragmentation may limit species dispersal, constraining postfire colonization dynamics and recovery potential.

In this study, we investigated how a wildfire that occurred in the Lost Pines during the summer of 2022 influenced the diversity and assemblage composition of flight-active beetles by comparing (i) historical (2011) and contemporary (2024) samplings and (ii) contemporary assemblages across a fire severity gradient and between burned and unburned sites. We also quantified vegetation structure to evaluate whether structural characteristics explain variation in beetle richness and abundance and how these effects compare to those of fire severity. By integrating historical disturbance-free baselines with postfire sampling, this study provides insight into how temporal turnover, fire severity, and vegetation structure jointly shape flight-active beetle assemblages in fragmented range-edge forests.

## Methods

### Study Site

Our research was conducted at the Stengl Lost Pines Biological Station (SLP), a 581-acre preserve that is part of the Texas Field Station Network managed by the University of Texas at Austin (UT-Austin). SLP is located in Bastrop County, Texas, approximately 40 miles east of Austin (30°5'14.492'' N, 97°10'25.463''W). The station supports mixed coniferous and hardwood forest stands, including *Juniperus virginiana* L. (Cupressales: Cupressaceae), *P. taeda*, *Q. stellata*, *Q. marilandica* Münchh. (Fagales: Fagaceae), and *Carya texana* Buckl. (Fagales: Juglandaceae), alongside a dense understory of native shrubs and vines such as *Ilex vomitoria* Ait. (Aquifoliales: Aquifoliaceae), *Callicarpa americana* L. (Lamiales: Lamiaceae), *Vaccinium arboreum* Marsh. (Ericales: Ericaceae), *Smilax bona-nox* L. (Liliales: Smilacaceae), and the more common *Smilax smallii* Morong. (Liliales: Smilacaceae). As part of the westernmost extent of *P. taeda* habitat, SLP represents a unique ecological transition zone between eastern pine forests and central Texas oak woodlands (Plowes and Gibson 2019). In August 2022, the Pine Pond Fire burned approximately 350 acres of SLP over 9 days. This event created a mosaic of fire severities across the landscape and provided a unique opportunity to study the effects of wildfire on the diversity and assemblage composition of flight-active beetles in an isolated *P. taeda* ecosystem. Prior to 2022, SLP had experienced only small wildfires confined to the southwestern corner of the station (less than 5 acres in 2011 and approximately 10 acres in 2015), making the Pine Pond Fire the first large wildfire affecting the study area in recent decades.

### Beetle Survey

We selected 8 sampling sites across SLP, covering a fire severity gradient and including both burned and unburned sites (hereafter referred to as “burn status”; Fig. 1). At each site, we deployed a cross-vane panel trap (AlphaScents, Oregon, United States) coated with Fluon to enhance capture efficiency (Hanks et al. 2018). Traps were hung approximately 2 m above ground on the nearest tree to the center of circular plots, where we previously assessed the vegetation (see below). Vegetation and woody debris (vines, branches) that interfered with trap placement were minimally cleared (0.5 m from trap) and discarded nearby, without accumulating into piles. Debris removal helps maintain trap efficiency by ensuring unobstructed interception surfaces, increasing the likelihood that flight-active beetles contact the trap and fall into the collection cup. It also reduces the accumulation of material within the cup that could facilitate escape of captured individuals (Dodds et al. 2024, Godwin et al. 2025).

**Fig. 1.**
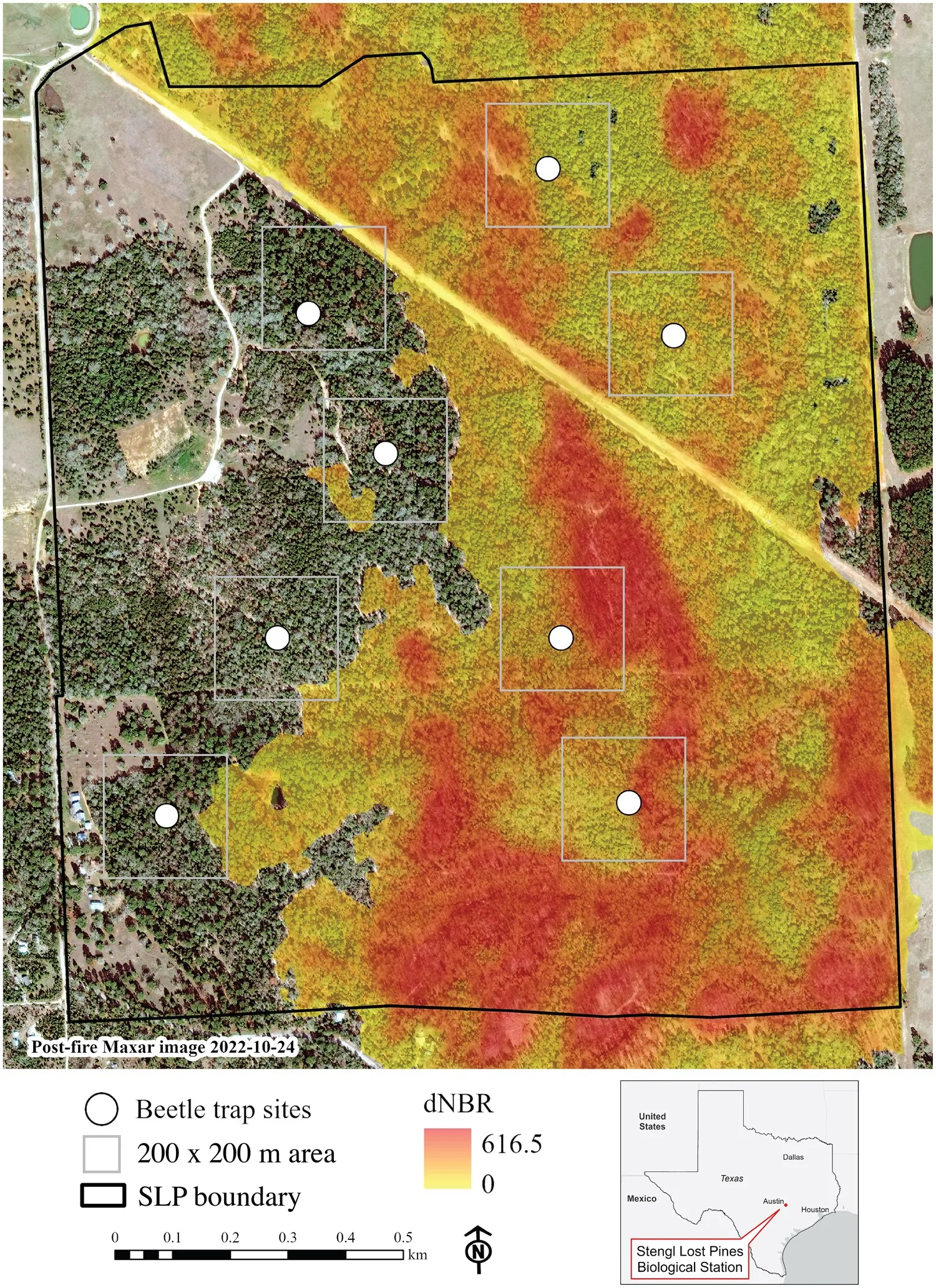
Map of the Stengl Lost Pines Biological Station, Texas, United States, showing the study area, sampling sites, and fire severity (dNBR) following the 2022 Pine Pond Fire. Circles indicate trap-scale reference sites and squares indicate local-scale reference sites. In the dNBR gradient, deeper, highly saturated, and darker warm red represents higher severity, and highly reflective, bright cool-toned yellow represents lower severity.

Each trap contained a collection cup filled with 400 ml of undiluted propylene glycol antifreeze. Lures consisted of a cotton ball soaked in a cerambycid pheromone blend enclosed in a plastic bag. The lures were prepared at the Hanks lab from the University of Illinois Urbana-Champaign and consisted of a blend designed by Fan et al. (2019) with 8 cerambycid pheromones. We chose these lures to match the methods used in the 2011 beetle survey conducted at SLP (30°5ʹ55.29"N, 97°9ʹ46.07"W; Hanks et al. 2018). Traps operated for an 8-week period between May and July 2024, with beetles collected weekly. This period represents the contemporary sampling and aligns with the 2011 sampling window (hereafter, historical sampling period). We replaced lures at the midpoint of the sampling period (after 4 weeks), and replenished antifreeze biweekly or after rainfall, following Hanks et al. (2018).

We acknowledge that the pheromone blend used was designed to attract Cerambycidae (Hanks et al. 2018, Fan et al. 2019), which may bias captures toward longhorn beetles and underrepresent other guilds. However, trap color and shape can attract a broader range of beetle taxa beyond the target group (Roques et al. 2023), as reflected in our capture of individuals from 22 families (see Results). We therefore interpret our results as represening patterns in the flight-active beetle assemblage captured by our traps rather than the broader beetle community.

The collected beetle specimens underwent a multistep laboratory process involving sorting, preservation, and identification. First, we rinsed the beetles to remove residual antifreeze and then pinned, identified, and curated the specimens to be submitted to the University of Texas Insect Collection. We consulted reference works such as Beetles of Eastern North America (Evans 2014) and online resources including iNaturalist, and we confirmed identifications with beetle taxonomists (Jason Hanson [Buprestid] and Tom Atkinson [*Xyleborus*]). To further validate our identifications, we submitted up to 3 specimens per morphospecies for molecular barcoding at the UT-Austin Brackenridge Field Laboratory.

### Beetle DNA Extraction and Barcoding

We extracted DNA using a DNeasy Blood and Tissue Kit (QIAGEN, Hilden, Germany) following the manufacturer Animal Tissue (Spin-Column) protocol modified for insect specimens. Legs were removed from specimens and crushed using a sterile pestle and soaked in 20 µl Proteinase K and 180 µl Buffer ATL at 56 °C for 24 h. Entire specimens were soaked without crushing if the specimen was small (<5 mm) and would not produce sufficient DNA yields with legs alone. Further DNA extraction protocol had no modification from the Animal Tissue (Spin-Column) protocol. Extracted DNA was stored at −20 °C until further use.

Extracted DNA underwent Polymerase Chain Reaction (PCR) amplification for the CO1 region using the LCO1490/HCO2198 primers (Folmer et al. 1994) and the CLepFolF/CLepFolR primers (Ivanova et al. 2007). DNA extracts were first tested with the LCO1490/HCO2198 primer pair. Samples that did not provide a quality sequence using the LCO1490/HCO2198 primers were sequenced again using the CLepFolF/CLepFolR primers. The 20 µl reactions consisted of 10 µl of Qiagen Multiplex PCR Mix (QIAGEN, Hilden, Germany), 5.6 µl PCR grade water (QIAGEN, Hilden, Germany), 2 µl of prepared 2 µM forward and reverse primer mix for respected primers, and 2.4 µl of extracted sample template. Cycling parameters for LCO1490/HCO2198 primers consisted of an initial denaturation of 95 °C for 5 min, followed by 35 cycles of 94 °C for 1 min, 50 °C for 1 min, and 72 °C for 1 min using the T100 Thermal Cycler (Bio-Rad, Hercules, California, United States). CLepFolF/CLepFolR cycling conditions were an initial denaturation of 95 °C for 15 min, followed by 5 cycles of 94 °C for 30 s, 45 °C for 40 s, and 72 °C for 1 min, followed by 35 cycles of 94 °C for 30 s, 50 °C for 40 s, and 72 °C for 1 min using T100 Thermal Cycler (Bio-Rad, Hercules, California, United States). Each run consisted of both positive and negative controls. Negative controls were PCR grade water (QIAGEN, Hilden, Germany). PCR product was then sent to Eton Biosciences (Research Triangle Park, North Carolina, United States) for Sanger sequencing. Forward and reverse reads were aligned, and low-quality base calls were trimmed using Geneious Prime version 2024.0 (Biomatters 2024). Consensus sequences were created from the alignments and then passed through the NCBI nucleotide database using BLASTn (Basic Local Alignment Search Tool; Altschul et al. 1990). The top BLASTn result was assigned to a sample as the molecular identity. A match >99% was assigned to a species level. A match between 98% and 99% was assigned to a genus level, and matches between 94% and 98% were assigned to a family level. Consensus sequences have been submitted to GenBank (SUB16178216).

### Vegetation Structure Characteristics

We conducted vegetation surveys at each of the beetle sampling sites during the contemporary sampling period. These sites are part of 226 permanent vegetation plots at SLP. Each permanent plot included a circular area with a 7.3 m radius, where we identified, measured, tagged, and assessed all trees ≥7 cm diameter at breast height (DBH) for mortality status (live or dead). In addition, we surveyed four 2 × 2 m quadrats within each circular plot for shrubs and vines taller than 75 cm. We estimated tree and shrub density as the number of trees or shrubs per square meter. Abundance and richness were recorded as raw counts. Based on these, we estimated the Shannon diversity index for woody plants, and for trees and shrubs separately. Additionally, we estimated specific abundance (total, live, and dead) for the most abundant tree species: *P. taeda*, *J. virginiana*, *Q. stellata*, and *Q. marilandica*.

### Fire Severity

We calculated the normalized burn ratio (NBR) (Key and Benson 1999; 2006) and differential normalized burn ratio (dNBR). We used 20 m resolution Sentinel-2 imagery from the closest dates available pre- and postfire, 10 July and 28 September 2022, respectively (Copernicus Sentinel-2). The NBR was calculated from the visible–near infrared (VNIR; band 8,832 nm) and short-wave infrared (SWIR; band 122,202.4 nm) reflectances. Fire severity was estimated as the dNBR, calculated by subtracting the postfire NBR from the prefire NBR (Key and Benson 2006). For each trap, we assigned 2 measures of fire severity. First, we extracted trap-scale dNBR from the pixel containing the trap, providing a fine-scale estimate. Second, we calculated local-scale dNBR as the mean dNBR of all pixels within an approximately 200 × 200 m buffer surrounding the trap (Fig. 1), providing a broader-scale estimate of fire severity. In both cases, higher positive dNBR values indicate areas most severely affected by fire, while values near zero or negative indicate little or no effect (Hayes and Robeson 2009). Sentinel-2 provided the finest spatial resolution currently available that includes both NIR and SWIR bands, allowing a reasonable approximation of fire severity at both the trap and landscape scales.

### Statistical Analysis

We used the iNEXT package from R (Chao et al. 2014, Hsieh et al. 2016) to estimate and compare flight-active beetle diversity across sampling periods (historical vs. contemporary) and between burn status (burned vs. unburned; Fredriksson et al. 2020, Gutowski et al. 2020). iNEXT generated diversity estimates using incidence-based data and constructed rarefaction and extrapolation curves based on sample size, coverage, and completeness. We calculated Hill numbers for *q* = 0 (richness), *q* = 1 (Shannon diversity), and *q* = 2 (Simpson diversity); the latter 2 emphasize common and dominant species, respectively (Hill 1973, Chao and Jost 2012, Hsieh et al. 2016, Roswell et al. 2021).

As mentioned previously, we used SLP’s historical beetle data from 2011 (Hanks et al. 2018) for comparing flight-active beetle assemblages between sampling periods. Because the historical sampling targeted wood-boring and predatory taxa, we restricted our analyses to these guilds when comparing historical and contemporary datasets. We refer to this subset as the wood-associated assemblage. We emphasize that this historical dataset represents a disturbance-free reference rather than an immediate prefire snapshot.

We compared flight-active beetle assemblage composition between sampling periods and between burn status using a Bray–Curtis distance matrix based on species abundances, followed by nonmetric multidimensional scaling (NMDS) to visualize assemblage patterns. To test for differences in assemblage composition, we conducted a permutational multivariate analysis of variance (PERMANOVA) with 999 permutations (*P *< 0.05) and evaluated homogeneity of multivariate dispersion using a beta-dispersion test. To account for unequal sample sizes between sampling periods (36 in the historical sampling and 8 in the contemporary sampling, or 4 if subset by burn status), we conducted a Monte Carlo subsampling analysis in which the historical samples were randomly subsampled to match the contemporary sample size across 1,000 iterations.

Using the contemporary data, we examined how vegetation structural characteristics and fire severity—at both dNBR_trap and dNBR_local levels—influence flight-active beetle assemblages. We fit a series of generalized linear models (GLMs) using the function *glm* with a Poisson error distribution and log-link function separately for beetle richness and abundance. All predictors were *z*-transformed prior to analysis to aid model convergence and comparability of coefficients. We also checked for variables multicollinearity with the *vif* function from the car package (Fox and Weisberg 2019) and removed multicollinear predictors from each model.

We then adopted a stepwise modeling approach. First, we fit models with general woody plant (WP) variables (total WP richness, abundance, and diversity) along with our measure of fire severity (dNBR_trap or dNBR_local) to compare the influence of fire versus vegetation structure. Because WP metrics included both tree and shrub components, we then ran models that separated trees and shrubs into distinct variables (tree richness, tree density, tree diversity, mean tree DBH, shrub richness, shrub density, and shrub diversity). Next, to test whether tree survival status provided additional explanatory power, we partitioned tree density and DBH into live and dead components. Finally, to evaluate the role of dominant tree taxa at SLP, we constructed 2 taxon-specific models: one using densities of live *P. taeda*, *J. virginiana*, and *Quercus* spp.; and one using densities of dead individuals of the same taxa. The following predictors: dNBR (trap or local), shrub richness, and shrub density were retained in all models. For each modeling stage, we used stepwise Akaike Information Criterion (AIC) selection (both forward and backward) to identify the most parsimonious model. We examined model summaries to assess the direction and magnitude of predictor effects. All analyses were conducted in R version 4.4.2 (R Core Team 2024).

## Results

We collected a total of 570 beetle specimens representing the flight-active beetle assemblage during the 8-week contemporary sampling period. Across 9 collection dates, we captured 77 morphospecies representing 22 families. Based on combined morphological and DNA barcoding methods, we identified 68.8% of the morphospecies to species, 19.5% to genus, 2.3% to subfamily, and 9.1% to family level (Supplementary Table S1).

DNA barcoding provided taxonomic information for 40 morphospecies (51.9%), whereas 35 (45.5%) morphospecies had low sequence similarity or unresolved results; 2 morphospecies were not submitted for barcoding analysis. Of the 40 morphospecies with informative barcode results, 13 were assigned to species level based on a >99% match. Six additional morphospecies had >99% matches to multiple taxa, to lower taxonomic levels, or to unclassified organisms and therefore did not yield an unambiguous species-level assignment. Thirteen morphospecies were assigned to genus level based on matches between >98% and 99%, and 8 were assigned to family level based on matches between 94% and 98%. Unresolved or discordant barcode assignments have also been reported in insect barcoding studies and may reflect incomplete reference-database coverage, multiple taxa sharing similar barcode sequences, geographic variation in barcode sequences, or other limitations of sequence-based identification (Pentinsaari et al. 2014, Salis et al. 2024). Because we did not independently evaluate the causes of unresolved sequences in our dataset, we cannot determine which factors contributed to individual cases. Overall, DNA barcoding contributed to the identification of 7 species, 4 genera, and 1 subfamily that could not be identified based on morphology alone.

Despite the pheromone blend being designed to target Cerambycidae (Hanks et al. 2018, Fan et al. 2019), and thus potentially biasing captures toward longhorn beetles (17 morphospecies), individuals from a broad range of taxa were collected, including Buprestidae (7 morphospecies), Carabidae (2), and Scarabaeidae (5). The assemblage also included a high proportion of rare taxa, with 40 morphospecies represented by a single individual, indicating a substantial contribution of singletons to overall contemporary diversity.

The Cerambycidae family was the most common, representing 264 specimens (46% of total specimens). The 4 most abundant species—cerambycids (Coleoptera: Cerambycidae) *Neoclytus acuminatus* (Fabricius) (135 specimens, 24%), *Lepturges angulatus* (LeConte) (48 specimens, 8%), clerid (Coleoptera: Cleridae) *Chariessa pilosa* (Forster) (49 specimens, 9%), and tenebrionid (Coleoptera: Tenebrionidae) *Lobopoda* sp. (40 specimens, 7%)—collectively accounted for nearly half (48%) of all specimens collected.

In the vegetation survey, we recorded 4 species of trees, with *Q. stellata* being the most abundant one (33), followed by *J. virginiana* (31), *P. taeda* (12), and *Q. marilandica* (10). For statistical analysis, we merged the 2 *Quercus* species into a single group because they share similar ecological traits and functional roles in postfire forest dynamics. Among shrubs, we recorded 5 species, with *I. vomitoria* being the most dominant (118), followed by *S. bona-nox* (44), *C. americana* (7), *S. smallii* (3), and *Sideroxylon lanuginosum* Michx., 1803 (Ericales: Sapotaceae; 1).

### Beetle Diversity and Assemblage Composition across Sampling Periods

We collected 378 specimens representing 38 species classified as wood borer or predatory beetle species during the contemporary sampling period, with a capture rate of 47.4 individuals per trap. In contrast, the historical sampling recorded 366 specimens from 20 wood borer or predatory species, with a capture rate of 10.2 individuals per trap. The most frequently collected species in the historical sampling was *N. acuminatus* with 77 specimens (21%) followed by *C. pilosa* (54, 15%), *Monochamus carolinensis* (Olivier) (50, 14%, Coleoptera: Cerambycidae), and *N. mucronatus* (Fabricius) (48, 13%). Similarly, in the contemporary sampling, *N. acuminatus* was the most abundant species with 135 specimens (36%) followed by *C. pilosa* (49, 13%), *L. angulatus* (48, 12.7%), and *Eustrophopsis bicolor* (Fabricius) (23, 6%).

Sample coverage was high in both sampling periods (historical 98.9% vs. contemporary 95.8%), indicating that sampling captured most of the wood-associated assemblage in each period. Despite similar coverage, observed species richness was nearly twice as high in the contemporary sampling (38 species) compared to the historical sampling (20 species; Fig. 2A). Patterns in diversity metrics, however, revealed contrasting trends. Hill–Shannon diversity was slightly higher in the contemporary period (11.2) than in the historical one (10.6), whereas Hill–Simpson diversity was higher in the historical period (8.3 vs. 5.8) (Table 1; Fig. 2A). Together, these results suggest that although richness increased in the contemporary assemblage, evenness declined, with wood-associated communities becoming increasingly dominated by a subset of species.

**Fig. 2.**
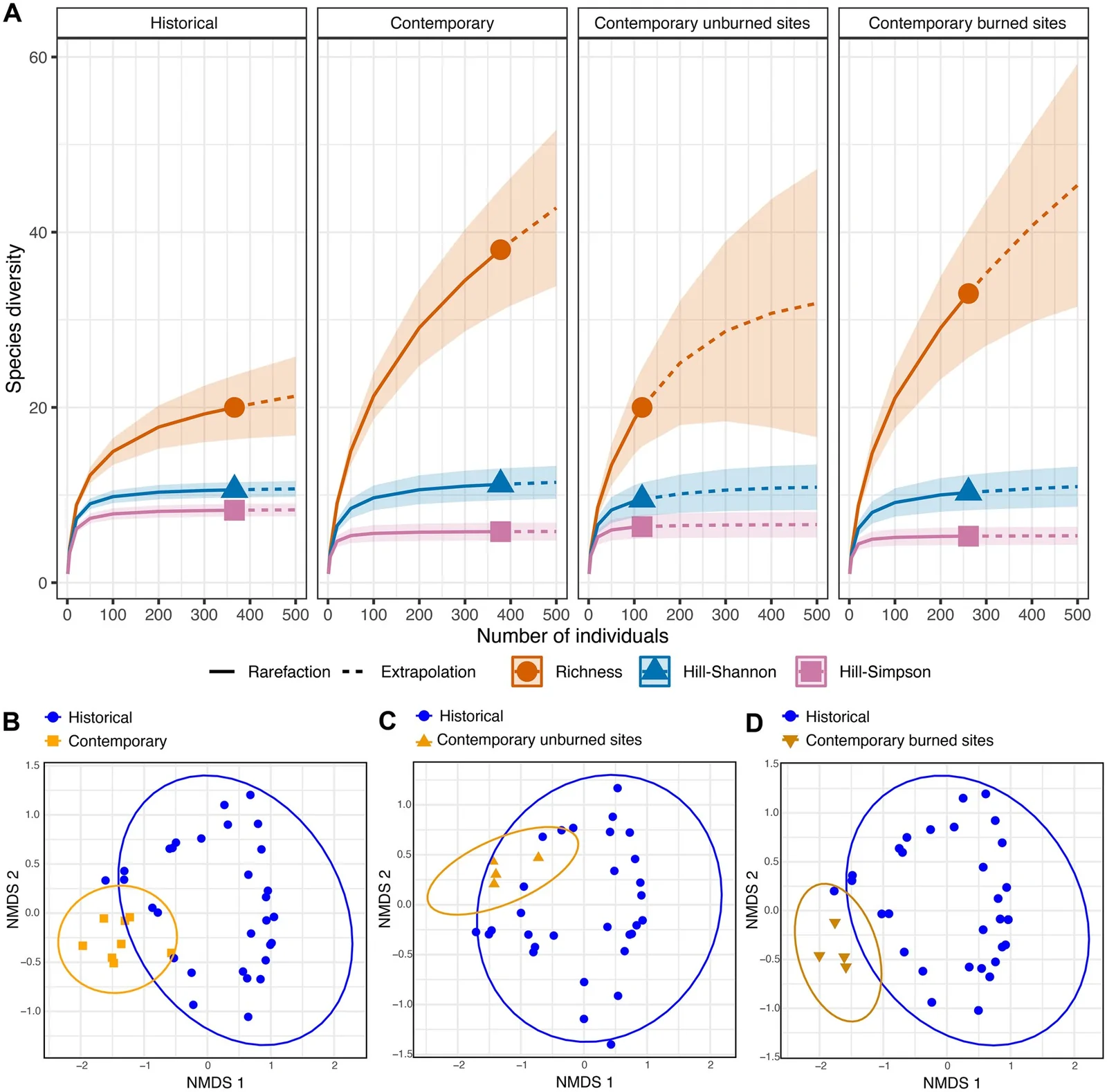
A) Incidence-based rarefaction/extrapolation curves for species richness, Hill–Shannon diversity and Hill–Simpson diversity comparing the historical sampling (2011), the contemporary sampling (2024), and contemporary unburned and burned sites. Shaded areas indicate 95% CI. B–D) NMDS of beetle community composition comparing the historical (2011) and contemporary (2024) samplings (B), the historical and contemporary unburned sites (C), and the historical and contemporary burned sites (D). Ellipses indicate 95% CI.

**Table 1.** Diversity metrics summary for historical and contemporary sampling (wood-associated community), and subsets of contemporary sites within unburned and burned areas

Dataset	Sampling units	Abundance	Coverage	Richness	Hill–Shannon	Hill–Simpson
**Historical**	36	366	98.9%	20	10.6	8.3
**Contemporary**	8	378	95.8%	38	11.2	5.8
**Contemporary unburned sites**	4	117	92.6%	20	9.5	6.4
**Contemporary burned sites**	4	261	93.9%	33	10.3	5.3

When comparing historical sampling to contemporary unburned and burned sites, diversity patterns diverged by burn status. Burned sites exhibited substantially higher richness (33 species) than the historical assemblage, but lower Hill–Shannon and Hill–Simpson diversity. In contrast, unburned sites showed richness comparable to historical levels (20 species), but also lower diversity values overall. The magnitude of change was greater in burned sites, suggesting a stronger decline in evenness relative to both historical and unburned assemblages (Table 1; Fig. 2A).

Ordination of wood-associated assemblages showed partial separation between historical and contemporary sampling periods (NMDS stress value = 0.1; Fig. 2B). PERMANOVA indicated a significant difference in overall assemblage composition ($F_{1,43}$ = 7.69, *R*² = 0.15, *P *= 0.001), but the beta-dispersion test revealed unequal multivariate dispersion between periods (*P *= 0.002), suggesting differences in within-group variability. The subsampling sensitivity analysis showed that across iterations, PERMANOVA effect sizes were consistent (mean *R*² = 0.30; median = 0.30; 95% CI = 0.19 to 0.44), and all iterations yielded significant differences between sampling periods (*P *< 0.05). These results indicate that temporal differences in wood-associated community composition are robust to sampling imbalance, despite heterogeneity in dispersion.

When comparing historical and contemporary assemblages within burn status, separation was more pronounced between historical and contemporary burned sites (NMDS stress value = 0.10; Fig. 2D) than between historical and contemporary unburned sites (NMDS stress value = 0.10; Fig. 2C). In both cases, PERMANOVA indicated significant differences in composition (burned: $F_{1,39}$ = 5.3, *R*² = 0.12, *P *= 0.001; unburned: $F_{1,39}$ = 3.9, *R*² = 0.09, *P *= 0.001), but dispersion also differed significantly (*P *= 0.001). Subsampling sensitivity analyses revealed that the comparison with burned sites was more robust, with 84% of iterations yielding significant results and consistently high effect sizes (mean *R*² = 0.42; media = 0.41; 95% CI = 0.26 to 0.61). In contrast, the comparison with unburned sites was less stable, with 55% of iterations significant and more variable effect sizes (mean *R*² = 0.36; media = 0.34; 95% CI = 0.18 to 0.57).

These shifts in diversity metrics and assemblage composition indicate temporal turnover in wood-associated community structure even in the absence of fire. Temporal turnover in community composition was more pronounced and consistent in burned sites than in unburned sites, suggesting that fire history amplifies flight-active beetle community reorganization over time.

### Fire Effects on the Diversity and Assemblage Composition of Flight-Active Beetles

Sample coverage was similar between burned and unburned sites (90.6% vs. 91.8%), indicating comparable sampling completeness between burn status. We captured a greater number of individuals in burned sites (343) than in unburned sites (227). The most frequently collected species in the burned sites was *N. acuminatus* with 100 specimens (29.4%) followed by *C. pilosa* (34, 9.9%), *L. angulatus* (30, 8.7%), and *Eustrophopsis bicolor* (22, 6.4%). Whereas the most frequently collected morphospecies in unburned sites was *Lobopoda* sp. (37, 16.3%), followed by *N. acuminatus* (35, 15.4%), and *Melanotus* sp. (23, 10.1%, Coleoptera: Elateridae).

We also observed higher richness in burned sites (64 vs. 40 morphospecies; Fig. 3A); however, diversity metrics revealed contrasting patterns. Hill–Shannon diversity was slightly higher in burned sites (20.56 vs. 19.23), whereas Hill–Simpson diversity was higher in unburned sites (12.32 vs. 8.66), suggesting differences in dominance structure between burn status (Fig. 3A).

**Fig. 3.**
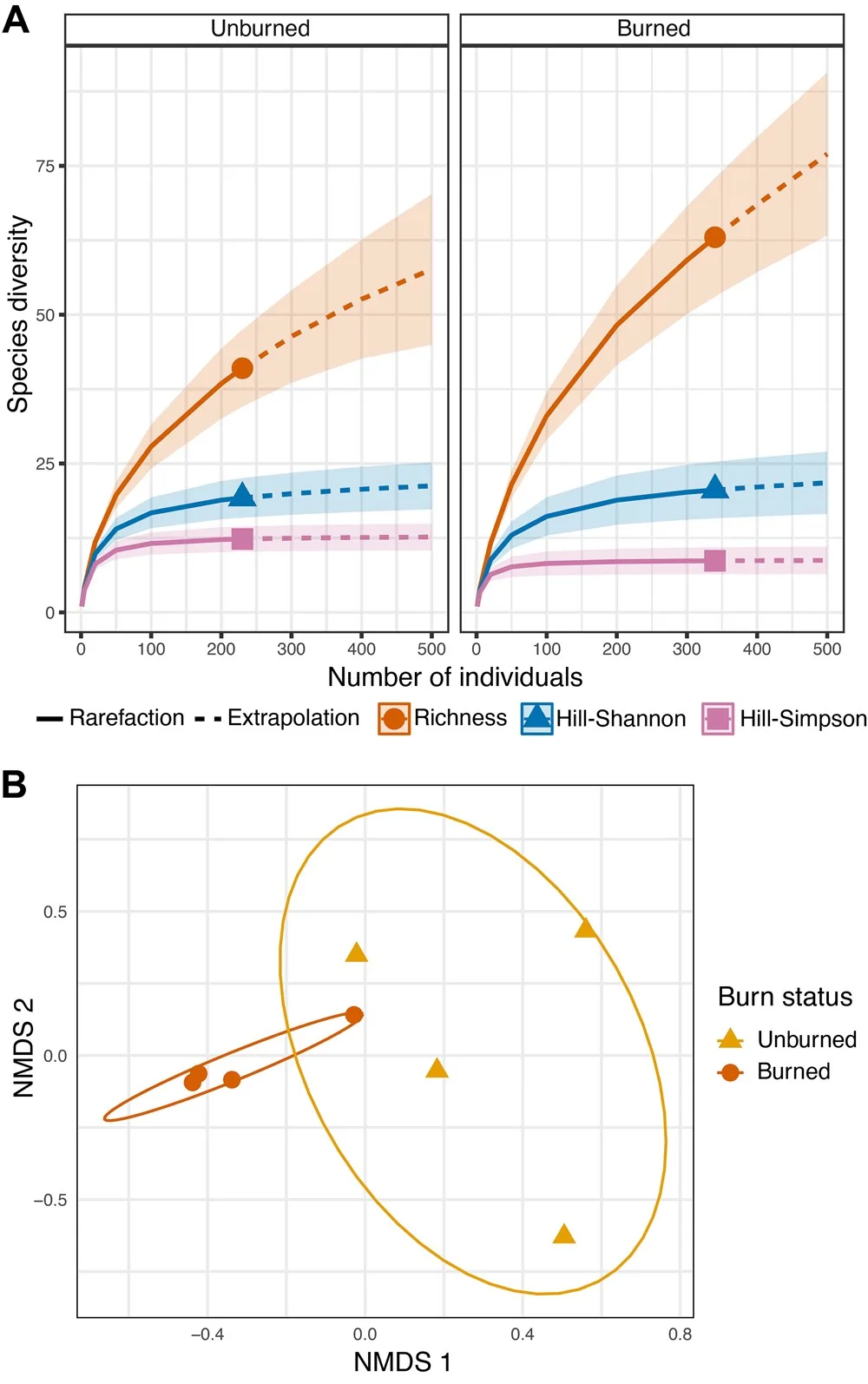
A) Incidence-based rarefaction/extrapolation curves for species richness, Hill–Shannon diversity and Hill–Simpson diversity comparing the contemporary (2024) unburned and burned sites. Shaded areas indicate 95% CI. B) NMDS of beetle community composition across burn status (2024). Ellipses indicate 95% CI.

Despite these differences, NMDS ordination showed some overlap in flight-active beetle assemblage composition between burned and unburned sites (stress value = 0.02, Fig. 3B). Consistent with this pattern, PERMANOVA indicated a marginally non-significant difference in assemblage composition ($F_{1,7}$ = 1.72, *P *= 0.06), while the beta-dispersion test showed no difference in multivariate dispersion (*P *= 0.92). We acknowledge that interpretation of composition differences between burned and unburned assemblages is constrained by low replication (*n* = 4). The marginally non-significant difference detected here, coupled with overlapping NMDS patterns and no difference in dispersion, suggests that any compositional differences between burned and unburned sites are subtle and should be interpreted cautiously until validated by studies with greater replication.

### Fire Severity and Vegetation Structure Effects on Beetle Diversity

Results of all models are reported in Tables 2 and 3. Using trap-scale dNBR, beetle richness showed a consistent positive association with fire severity across most models (Models R-1, R-3, R-4a, R-4b; *P *= 0.005 to 0.030). In the initial model (Model R-1), richness increased with both fire severity (*P *= 0.018) and WP richness (*P *= 0.045). Subsequent models incorporating vegetation structure retained the positive effect of fire severity, while vegetation variables showed weaker and less consistent effects. Notably, richness was positively associated with shrub richness in Model R-4b (*P *= 0.030) and negatively associated with dead *J. virginiana* density (*P *= 0.037).

**Table 2.** Results of GLMs selected during stepwise model selection for beetle richness and abundance using trap-scale dNBR

Model	Estimate	SE	***z***	Pr(<|*z*|)	
**Beetle richness**					
**Model R-1**					
***Richness ∼ dNBR_trap + WP richness***					
**(Intercept)**	3.10653	0.07559	41.096	<0.001	***
**dNBR_trap**	0.17737	0.07554	2.348	0.018	*
**WP richness**	0.15463	0.07713	2.005	0.045	*
**Model R-2**					
***Richness ∼ dNBR_trap + shrub richness***					
**(Intercept)**	3.11117	0.07523	41.353	<0.001	***
**dNBR_trap**	0.14135	0.07763	1.821	0.069	.
**Shrub richness**	0.11798	0.07472	1.579	0.114	
**Model R-3**					
***Richness ∼ dNBR_trap + live tree density + shrub richness***					
**(Intercept)**	3.10457	0.07577	40.976	<0.001	***
**dNBR_trap**	0.18472	0.08343	2.214	0.027	*
**Live tree density**	0.12386	0.08297	1.493	0.136	
**Shrub richness**	0.13160	0.07616	1.728	0.084	.
**Model R-4a**					
***Richness ∼ dNBR_trap + live Quercus density + shrub density***					
**(Intercept)**	3.10421	0.07594	40.876	<0.001	***
**dNBR_trap**	0.24121	0.08608	2.802	0.005	**
**Live *Quercus* density**	0.14381	0.08476	1.697	0.089	.
**Shrub density**	0.14490	0.09218	1.572	0.116	
**Model R-4b**					
***Richness ∼ dNBR_trap + dead J. virginiana density + dead Quercus density + shrub richness***					
**(Intercept)**	3.09617	0.07655	40.446	<0.001	***
**dNBR_trap**	0.23037	0.10620	2.169	0.030	*
***Dead J. virginiana density***	−0.33813	0.16191	−2.088	0.037	*
***Dead Quercus density***	0.31097	0.19085	1.629	0.103	
**Shrub richness**	0.30727	0.14175	2.168	0.030	*
**Beetle abundance**					
**Model A-1**					
***Abundance ∼ dNBR_trap + WP richness***					
**(Intercept)**	4.24331	0.04281	99.113	<0.001	***
**dNBR_trap**	0.12826	0.04361	2.941	0.003	**
**WP richness**	0.19378	0.04426	4.378	<0.001	***
**Model A-2**					
***Abundance ∼ dNBR_trap + tree diversity + mean tree DBH + shrub richness + shrub density***					
**(Intercept)**	4.22789	0.04352	97.156	<0.001	***
**dNBR_trap**	0.16507	0.06246	2.643	0.008	**
**Tree diversity**	0.26687	0.06262	4.262	<0.001	***
**Mean tree DBH**	0.30382	0.07255	4.188	<0.001	***
**Shrub richness**	0.11281	0.05835	1.933	0.053	.
**Shrub density**	−0.16060	0.07885	−2.037	0.042	*
**Model A-3**					
***Abundance ∼ dNBR_trap + live tree density + shrub richness***					
**(Intercept)**	4.24630	0.04270	99.455	<0.001	***
**dNBR trap**	0.11820	0.04830	2.447	0.014	*
**Live tree density**	0.09903	0.04678	2.117	0.034	*
**Shrub richness**	0.15751	0.04397	3.583	<0.001	***
**Model A-4a**					
***Abundance ∼ dNBR_trap + live P. taeda density + live Quercus density + shrub density***					
**(Intercept)**	4.24471	0.04278	99.219	<0.001	***
**dNBR_trap**	0.13473	0.05175	2.604	0.009	**
**Live *P. taeda* density**	0.11935	0.04400	2.713	0.006	**
**Live *Quercus* density**	0.13434	0.04578	2.935	0.003	**
**Shrub density**	0.09948	0.05138	1.936	0.053	.
**Model A-4b**					
***Abundance ∼ dNBR_trap + shrub density***					
**(Intercept)**	4.25783	0.04223	100.814	<0.001	***
**dNBR_trap**	0.13840	0.04540	3.049	0.002	**
**Shrub density**	0.07009	0.04737	1.480	0.139	

Significance codes: 0 “***” 0.001 “**” 0.01 “*” 0.05 “.” 0.1 “ ” 1.

**Table 3.** Results of GLMs selected during stepwise model selection for beetle richness and abundance using local-scale dNBR (200 × 200 m)

Model	Estimate	SE	***z***	Pr(<|*z*|)	
**Beetle richness**					
**Model R-5**					
***Richness ∼ dNBR_local + WP richness***					
**(Intercept)**	3.10650	0.07556	41.112	<0.001	***
**dNBR_local**	0.17958	0.07512	2.391	0.017	*
**WP richness**	0.11146	0.07881	1.414	0.157	
**Model R-6**					
***Richness ∼ dNBR_local***					
**(Intercept)**	3.11160	0.07522	41.366	<0.001	***
**dNBR_local**	0.20017	0.07385	2.711	0.007	**
**Model R-7**					
***Richness ∼ dNBR_local + live tree density + shrub density***					
**(Intercept)**	3.09575	0.07657	40.432	<0.001	***
**dNBR_local**	0.30309	0.09002	3.367	<0.001	***
**Live tree density**	0.18759	0.09157	2.049	0.040	*
**Shrub density**	0.13585	0.09100	1.493	0.135	
**Model R-8a**					
***Richness ∼ dNBR_local + live Quercus density + shrub density***					
**(Intercept)**	3.09743	0.07643	40.529	<0.001	***
**dNBR_local**	0.26917	0.08325	3.233	0.001	**
**Live *Quercus* density**	0.16289	0.08472	1.923	0.055	.
**Shrub density**	0.12552	0.08849	1.418	0.156	
**Model R-8b**					
***Richness ∼ dNBR_local + dead J. virginiana density + dead Quercus density + shrub richness***					
**(Intercept)**	3.09434	0.07663	40.380	<0.001	***
**dNBR_local**	0.28478	0.11979	2.377	0.017	*
**Dead *J. virginiana* density**	−0.35765	0.16186	−2.210	0.027	*
**Dead *Quercus* density**	0.29850	0.18926	1.577	0.115	
**Shrub richness**	0.21541	0.14792	1.456	0.145	
**Beetle abundance**					
**Model A-5**					
***Abundance ∼ dNBR_local + WP richness***					
**(Intercept)**	4.23991	0.04293	98.772	<0.001	***
**dNBR_local**	0.15728	0.04274	3.680	<0.001	***
**WP richness**	0.15710	0.04515	3.470	<0.001	***
**Model A-6**					
***Abundance ∼ dNBR_local + tree diversity + mean tree DBH***					
**(Intercept)**	4.22528	0.04366	96.768	<0.001	***
**dNBR_local**	0.27052	0.05092	5.313	<0.001	***
**Tree diversity**	0.27036	0.06012	4.497	<0.001	***
**Mean tree DBH**	0.20542	0.04729	4.344	<0.001	***
**Model A-7**					
***Abundance ∼ dNBR_local + live tree density + dead tree density + shrub richness***					
**(Intercept)**	4.23630	0.04316	98.154	<0.001	***
**dNBR_local**	0.27437	0.06876	3.990	<0.001	***
**Live tree density**	0.12197	0.04747	2.569	0.010	*
**Dead tree density**	−0.12051	0.06658	−1.810	0.070	.
**Shrub richness**	0.07484	0.04857	1.541	0.123	
**Model A-8a**					
***Abundance ∼ dNBR_local + live Quercus density + shrub density***					
**(Intercept)**	4.23603	0.04320	98.063	<0.001	***
**dNBR_local**	0.25557	0.04722	5.413	<0.001	***
**Live *Quercus* density**	0.16759	0.04747	3.530	<0.001	***
**Shrub density**	0.11626	0.05007	2.322	0.020	*
**Model A-8b**					
***Abundance ∼ dNBR_local + dead P. taeda density***					
**(Intercept)**	4.24688	0.04265	99.568	<0.001	***
**dNBR_local**	0.25492	0.05314	4.797	<0.001	***
**Dead *P. taeda* density**	−0.10654	0.05286	−2.015	0.044	*

Significance codes: 0 “***” 0.001 “**” 0.01 “*” 0.05 “.” 0.1 “ ” 1.

Beetle abundance exhibited a similar overall pattern. In the initial model (Model A-1), abundance increased with both fire severity (*P *= 0.003) and WP richness (*P *< 0.001), and the positive effect of fire severity remained significant across all models (Models A-2 to A-4b). When vegetation structure was partitioned (Model A-2), abundance increased with tree diversity and mean tree DBH (both *P *< 0.001) but decreased with shrub density (*P *= 0.042). Models incorporating live and dead tree variables further indicated that live tree variables—particularly live *P. taeda* and *Quercu*s trees—were positively associated with abundance, whereas dead tree variables were not retained. In contrast to shrub density, shrub richness was positively associated with abundance (Model A-3, *P *< 0.001).

Together, these trap-scale results suggest that beetle richness and abundance are promoted by fire severity and by structurally complex forests characterized by diverse and large live trees, as well as diverse shrub assemblages; whereas dense shrub cover may locally constrain beetle abundance.

Using local-scale dNBR, beetle richness again showed a positive and statistically significant association with fire severity across all models (Models R-5 to R-8b; *P *= 0.001 to 0.017). Compared to trap-scale models, the effect of fire severity was stronger and more consistent, even having models where fire severity alone explained variation in richness (Model R-5, 6, 8a; *P *= 0.001 to 0.017). Vegetation covariates were less frequently retained, with live tree density showing a significant positive effect (Model R-7; *P *= 0.040), and as in trap-scale models, dead *J. virginiana* density was negatively associated with richness (Model R-8b; *P *= 0.027). Shrub variables were not significant predictors of richness at this scale.

Beetle abundance also showed strong and consistent positive associations with fire severity across all models (Models A-5 to A-8b; all *P *< 0.001). Vegetation structure variables exhibited patterns similar to those observed at the trap-scale: abundance increased with WP richness (Model A-5; *P* = <0.001), tree diversity, mean tree DBH (Model A-6; both *P *< 0.001), and live tree density (Model A-7; *P *= 0.010), particularly live *Quercus* density (Model A-8a; *P *< 0.001). In this case, dead *P. taeda* density was negatively associated with abundance (Model A-8b; *P *= 0.044). For shrub variables, only shrub density was a significant predictor, but it showed the opposite effect at this scale compared to trap-scale models, exhibiting a positive association with abundance in Model A-8a (*P *= 0.020).

## Discussion

By incorporating rare historical data as a disturbance-free baseline for the SLP flight-active beetle community, our study allows direct comparison of beetle assemblages across sampling periods (historical [2011] vs. contemporary [2024]), rather than relying solely on postfire patterns. Using this framework, we detected a clear temporal turnover in beetle communities over this 13-year period, alongside strong responses to wildfire and a secondary role of postfire vegetation structure. Together, these processes shaped richness, abundance, and evenness, indicating that both long-term temporal change and disturbance interact to structure community dynamics.

Within wood-associated beetle assemblages, richness and abundance were higher in the contemporary than in the historical sampling period, suggesting substantial background community reorganization over time. This temporal turnover appears to be partially independent of fire, likely reflecting broader environmental changes occurring over the intervening decade. However, these temporal shifts were amplified within the burned area, where wildfire imposed an additional ecological filter that further reshaped community composition and increased divergence from the historical assemblage. Despite increases in richness and abundance in contemporary samples, evenness declined, indicating increasing dominance by a few opportunistic species. Similar decadal temporal turnover has been documented in other systems, including dung beetles over a 17-year interval in a Neotropical rainforest (Salomão et al. 2020).

Richness and abundance of flight-active beetles were higher in contemporary burned than in unburned sites, consistent with the strong fire effect superimposed on the broader background temporal turnover. Increased postfire richness and abundance have been widely reported in beetle communities across temperate and boreal forests (Hyvärinen et al. 2006, Fredriksson et al. 2020, Lawhorn et al. 2025), often driven by the rapid colonization of pyrophilous and other disturbance-adapted species typically within the first few years after fire (Saint-Germain et al. 2004, Moretti et al. 2004, Fredriksson et al. 2020, Lawhorn et al. 2025). Wood-boring and predatory beetles were more diverse in burned areas, highlighting the potential importance of postfire habitat heterogeneity, which likely provided newly available woody resources and associated prey. Although increased flowering following fire could also attract some flight-active beetles (Mason et al. 2021), the taxa contributing most strongly to the higher abundance in burned sites were predominantly wood-associated. Fire severity further influenced diversity patterns, with richness and abundance increasing consistently with severity. More intense fires likely generate greater spatial heterogeneity through canopy opening, altered microclimates, and increased variability in woody vegetation structure, thereby creating a wider range of ecological niches (Wikars 1997, Boulanger et al. 2010, Seibold et al. 2016).

In contrast, the decline in evenness observed in the contemporary sampling and in burned sites—now dominated by highly opportunistic species such as *N. acuminatus*, with an 8× increase in capture rate relative to the historical sampling—illustrates early successional dynamics characteristic of recently disturbed forests. This pattern is particularly evident in xylophagous assemblages, where generalist colonizers rapidly proliferate while more specialized or less mobile taxa recolonize more slowly (Bouget and Duelli 2004, Woodcock et al. 2012). The lower Hill–Simpson diversity in fire-affected assemblages likely indicates a community still in transition toward a more balanced composition. Similar transient dominance patterns have been documented for xylophagous beetles in postfire forests, such as temperate deciduous mixed forests in Kentucky (Lawhorn et al. 2025), pine-dominated boreal systems in Finland (Hyvärinen et al. 2006), and Araucaria–Nothofagus forests of the southern Andes (Tello et al. 2022), where early colonizers temporarily outcompete disturbance-sensitive species before assemblages stabilize through succession. Together, these results suggest that temporal turnover at SLP reflects both long-term background community change and short-term postfire successional dynamics driven by colonization processes and shifting habitats.

As noted previously, our study had some methodological limitations. The pheromone blend used in our survey was designed specifically to attract Cerambycidae, likely biasing captures toward longhorn beetles. This selectivity may help explain the strong dominance of the opportunistic cerambycid *N. acuminatus* in burned areas and limits our ability to characterize broader flight-active beetle assemblages. Future studies could address this limitation by incorporating additional trapping methods—such as unbaited flight-intercept traps, ethanol- or α-pinene–baited funnel traps, or even pitfall traps—to more effectively sample predators, decomposers, and herbivores. Combining complementary trap types would provide a more balanced assessment of community responses, capturing both early colonizers and disturbance-sensitive taxa.

Our results indicated that the richness and abundance flight-active beetles are strongly influenced by fire severity, with additional contributions from forest structural characteristics. Across both spatial representations of fire severity, beetle richness and abundance increased consistently with dNBR, highlighting the central role of wildfire shaping postfire beetle communities. This pattern supports the hypothesis that fire enhances habitat heterogeneity and resource availability (Moretti et al. 2004, Saint-Germain et al. 2004). The effect of fire severity was stronger and more consistent with models using the local-scale dNBR, in some cases explaining variation in richness independently of vegetation structure. This suggests that fire-driven processes operate at broader spatial scales than individual vegetation attributes and may override local structural effects.

Nonetheless, vegetation structure played an important, though secondary, role. Beetle abundance and richness increased with WP richness, tree diversity, mean tree DBH, live tree density, and shrub richness, indicating that structurally complex forests with diverse canopy and understory, and larger tress, support more abundant and richer beetle assemblages. These features likely increase substrate diversity, habitat continuity, and microclimatic stability, all of which are important for saproxylic and predatory beetles (Lassauce et al. 2011). In contrast, shrub effects were more nuanced: Shrub richness was positively associated with both richness and abundance, whereas shrub density showed context-dependent effects. At the trap-scale, dense shrub cover was negatively associated with abundance, suggesting that locally dense understories—at SLP, particularly of *I. vomitoria* (yaupon)—may reduce light availability and host plant diversity, limiting beetle activity despite high vegetation biomass (Traylor et al. 2022). However, in models using the local-scaled dNBR, shrub density was positively associated with abundance. Because shrub density was measured consistently across models, this shift likely reflects differences in model structure rather than a true scale-dependent ecological response. Specifically, the aggregation of fire severity may alter how variance is partitioned among predictors, such that shrub density captures broader habitat conditions (e.g. regeneration or productivity) in some models but local crowding effects in others. Together, these results suggest that shrub density does not exert a simple, independent effect on beetle abundance, but instead may reflect multiple, context-dependent ecological processes.

Partitioning tree variables revealed that live trees were consistently more important than dead trees in structuring postfire beetle communities. Beetle abundance increased with live tree density (general or by species), indicating that live trees provide essential canopy foliage, phloem, and bark resources. In contrast, dead tree density (general or by species) was generally not retained in models, and, when retained, was negatively associated with richness or abundance. These findings differ from studies emphasizing the importance of deadwood for saproxylic beetles (Seibold et al. 2016, Ulyshen 2016) and suggest that the contribution of fire-generated deadwood may be strongly time-dependent. Deadwood created by fire likely represents a transient resource pulse that is rapidly exploited during the earliest stages of postfire succession, with peak colonization occurring within months to the first year after disturbance (Saint-Germain et al. 2004, Boulanger et al. 2013). Because our sampling occurred approximately 2 years postfire, it likely captured communities after this initial colonization pulse had subsided. This interpretation is consistent with our broader evidence of temporal turnover, indicating that postfire flight-active beetle assemblages at SLP remain highly dynamic and continue to reorganize through time following disturbance. Anecdotally, we observed extensive beetle damage on dead *P. taeda* trees at SLP, supporting the idea that colonization of weakened or recently dead pines likely peaked earlier in the postfire succession than our sampling window captured.

Taxon-specific models further highlight the importance of tree species identity. Beetle richness declined with increasing dead *J. virginiana* density but tended to increase with live *Quercus* density. Beetle abundance increased with live *P. taeda* and *Quercus* densities and declined with increasing dead *P. taeda* density. The negative effects of *J. virginiana* likely reflect its dense, resinous wood, which is often unsuitable for saproxylic development and may release defensive compounds that deter colonization (McCullough et al. 1998, Gandhi and Herms 2010). In contrast, oaks and pines provide preferred substrates for fire-associated beetles, aligning with studies showing preferential colonization of fire-damaged oaks and pines in North American forests (Wikars 1997, Boulanger et al. 2010, Davis et al. 2012).

Overall, our findings demonstrate that flight-active beetle assemblages in this range-edge pine–oak ecosystem are shaped by the interaction between long-term temporal turnover and strong fire-driven successional dynamics. Fire severity emerged as the dominant driver, particularly at broader spatial scales, while vegetation structural characteristics modulated local habitat quality and influenced the persistence of diverse beetle assemblages through time. Structural complexity associated with large live trees, and diverse tree and shrub layers appears especially important for sustaining beetle diversity during postfire recovery. Management strategies that promote mixed tree assemblages, protect large surviving trees, and prevent dense shrub encroachment can enhance resource continuity and support both early-successional and later-recolonizing taxa (Boulanger et al. 2010, Stokland et al. 2012, Ulyshen 2016). In contrast, the role of deadwood appears more transient than expected, likely reflecting the rapid turnover of fire-associated resources and the temporally dynamic nature of saproxylic colonization. These results emphasize that postfire flight-active beetle communities have not remained static in response to disturbance, but are part of an ongoing successional trajectory shaped by both immediate fire effects and broader temporal community reorganization.

In the fragmented and climatically marginal Lost Pines, where repeated fires and drought stress increasingly interact with habitat fragmentation (Hampe and Petit 2005), conserving structural complexity and promoting mixed woody assemblages may help buffer insect biodiversity against ongoing and future environmental change. Expanding sampling approaches to include additional guilds and continuing long-term monitoring of diverse insect groups—as currently implemented at SLP for Coleoptera, Lepidoptera (moths, Gabbard et al. 2026), and Hymenoptera (Strobel et al. in press)—will be essential for fully understanding how temporal turnover and postfire succession dynamics shape insect communities.

## Supplementary Material

ieag101_Supplementary_Data
